# How to implement the framework for the elimination of mother-to-child transmission of HIV, syphilis, hepatitis B and Chagas (EMTCT Plus) in a disperse rural population from the Gran Chaco region: A tailor-made program focused on pregnant women

**DOI:** 10.1371/journal.pntd.0008078

**Published:** 2020-05-28

**Authors:** Favio Crudo, Pablo Piorno, Hugo Krupitzki, Analia Guilera, Constanza López-Albizu, Emmaria Danesi, Karerina Scollo, Susana Lloveras, Sebastián Mir, Marisa Álvarez, Silvio Yudis, Miguel Angel Cayo Fernández, Diego Cipri, Alejandro Krolewiecki, Ana Cristina Pereiro, María Victoria Periago, Marcelo Claudio Abril, Mariana Fernandez

**Affiliations:** 1 Fundación Mundo Sano, Buenos Aires, Argentina; 2 Asociación para el Desarrollo Sanitario Regional (ADESAR), San Antonio de Areco, Buenos Aires, Argentina; 3 Consejo Nacional de Investigaciones Científicas y Técnicas (CONICET), Centro de Educación Médica e Investigaciones Clínicas “Norberto Quirno” (CEMIC), Buenos Aires, Argentina; 4 Instituto de Parasitología “Dr. Mario Fatala Chaben,” Administración Nacional de Laboratorios e Institutos de Salud (ANLIS), Buenos Aires, Argentina; 5 Centro Nacional de Diagnóstico e Investigaciones en Endemo-epidemias (CeNDIE), ANLIS, Buenos Aires, Argentina; 6 Universidad Nacional de San Antonio de Areco, San Antonio de Areco, Buenos Aires, Argentina; 7 Ministerio de Salud Pública de Salta, Salta, Argentina; 8 Ministerio de Primera Infancia, Salta, Argentina; 9 Dirección de Salud, Gobierno Autónomo Regional del Gran Chaco, Bolivia; 10 XVI Región Sanitaria Boquerón, Ministerio de Salud Pública y Bienestar Social, Mariscal José Félix Estigarribia, Paraguay; 11 Consejo Nacional de Investigaciones Científica y Técnicas (CONICET), Instituto de Investigaciones en Enfermedades Tropicales (IIET), Universidad de Salta – Sede Regional Orán, San Ramón de Orán, Salta, Argentina; 12 Consejo Nacional de Investigaciones Científica y Técnicas (CONICET), Fundación Mundo Sano, Buenos Aires, Argentina; University of Washington, UNITED STATES

## Introduction

The framework for the elimination of mother-to-child transmission (EMTCT Plus) was proposed by the Pan American Health Organization in 2017 to all member states in order to widen the already existing framework for HIV and syphilis to include elimination of the infection with hepatitis B virus (HBV) and Chagas disease (ChD), now called EMTCT Plus. The objective of this wider initiative is to achieve and maintain the elimination of mother-to-child transmission (MTCT) of the infection with HIV, syphilis, ChD, and the perinatal infection by HBV as a public health problem, in line with the Strategy for Universal Access to Health and Universal Health Coverage [1]. The EMTCT Plus framework represents an interesting challenge for member states, since it requires adequate implementation strategies to overcome health system diversities. Additionally, each country implements this framework in a different manner and according to their own national administrative structure, which can even vary within each country in federal administrations. Moreover, available data from each country constitute global national figures that do not necessarily reflect regional variations. This is the case of many intervention areas with dispersed rural populations like the Tri-Border Area between Argentina, Bolivia, and Paraguay located in the Gran Chaco region. Moreover, this region is a hotspot for neglected tropical diseases [2], not only for intestinal helminth infections but also for ChD [3]. This is the area that will be used as an example in the current tutorial to aid others in the implementation of the framework. For this purpose, we have posed a series of statements that aim to describe the different components, based on our experience, that should be considered for implementation of this framework.

## Aims of the EMTCT Plus framework for the Region of the Americas

1. Reduction of the rate of MTCT of HIV to 2% or less
2. Reduction of the incidence of MTCT of syphilis (including stillbirths) to 0.5 cases or less per 1,000 live births
3. Reduction of hepatitis B antigen (HBsAg) prevalence among 4- to 6-year-old children to 0.1% or less
4. More than 90% of children cured of Chagas infection with posttreatment negative serology.

The prevention of MTCT of the infections included in the EMTCT Plus framework requires the application of different interventions directed specifically to women and their newborns before pregnancy and during pregnancy as well as after childbirth. Accordingly, a program for such an area needs to be designed bearing in mind the socioeconomic and environmental characteristics of the communities and centered on pregnant women and maternal-child health with a special focus on the opportunity of access to quality healthcare, the harmonization between the different levels of capacity of the health systems of the area, the concept of equity in access to health, and, ultimately, the strengthening of the healthcare capacities in the area in order to reach the proposed aims of EMTCT Plus (specific objectives are listed in Box 1). Since gender equality is a concept absolutely inherent to the context of the EMTCT Plus framework, the program needs to follow guidelines, norms, and practices related to sexual and reproductive health rights, women’s rights, and the health of their children.

**Box 1. Example of specific aims and objectives that can be formulated for a tailor-made program within the objectives of the EMTCT Plus framework: The case of the Tri-Border Area between Argentina, Bolivia, and Paraguay**

General program objective

Collaborate in the implementation of the EMTCT Plus framework in a geographical delimitated area of the Tri-Border Area between Argentina, Bolivia, and Paraguay in the Gran Chaco region together with the local, regional, and national health systems of each country in order to strengthen local capacity and promote access to high quality healthcare practices.

Specific program objectives

1. Identify the health services that are offered in the communities that are located in the area of the study in order to obtain baseline data.
2. Increase the coverage of pregnant women that receive checkups according to appropriate health practices to 100%.
  - Identify and treat infections by HIV, syphilis, HBV, and ChD in pregnant women and newborns
  - Identify high risk pregnancy for follow-up and eventual referral to more complex health centers, as needed
  - Increase the number of institutional births in the communities that live in the intervention area.
3. Optimize monitoring and follow-up of the quality of maternal-child health attention through the use of quality software to improve clinical management of those included in the program.
4. Evaluate efficacy of the sanitary intervention model proposed.

## Characteristics of areas of the Americas with dispersed rural populations that need to be considered for implementation

### Geographic and climatic characteristics

**Fig 1:**
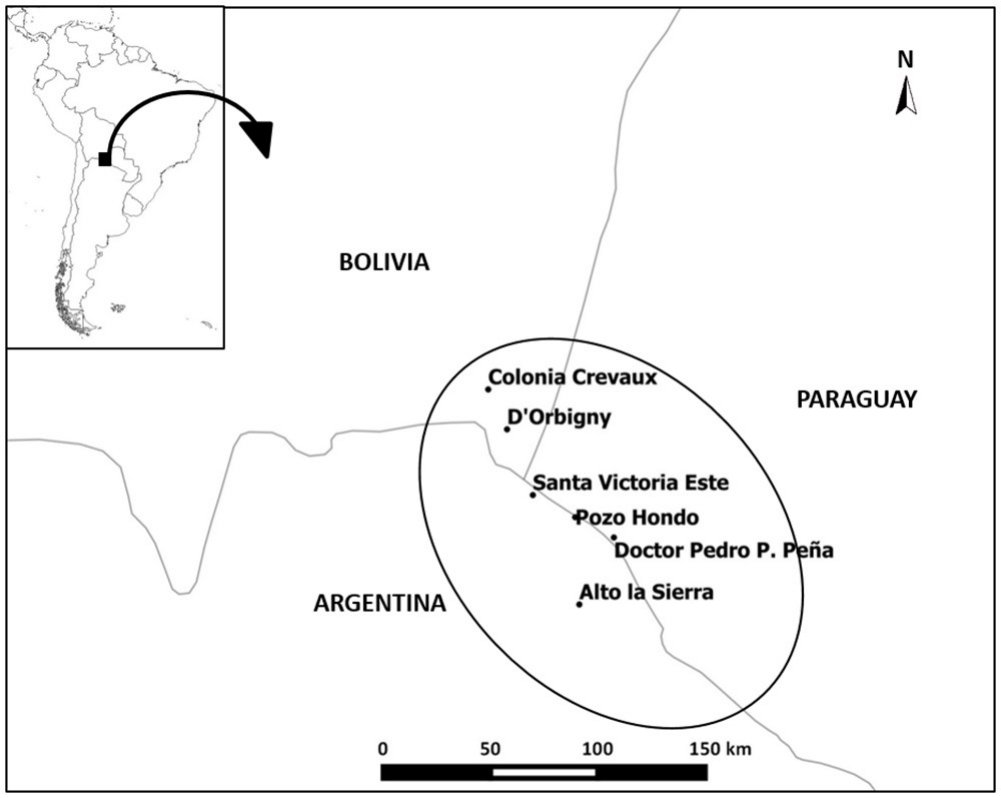
Map of the localities included in the intervention program. Santa Victoria Este and Alto la Sierra in Argentina, Colonia Crevaux and D´Orbigny in Bolivia, and Pozo Hondo and San Agustín/Doctor Pedro P. Peña in Paraguay. Map created with QGIS 2.4 open-source software.

In the case of the Tri-Border Area, the program is implemented in rural and semi-urban communities from the municipality of Santa Victoria Este and the locality of Alto la Sierra in Salta Province (Argentina), the localities of Creavaux and D’Orbigny and close rural communities from the Autonomous Region of Chaco (Bolivia), and the localities of Pozo Hondo and San Agustín/Doctor Pedro P. Peña as well as close rural communities from the Department of Boquerón (Paraguay) (Fig 1). This region is characterized for presenting a subtropical climate with a dry season between the months of April and December and a very rainy season during the rest of the year. The region is crossed from east to west by the Pilcomayo River, which frequently overflows and causes floods, which lead to the isolation of the population living in the area.

### Population demographics and cultural characteristics

The population living in the area of intervention, according to the census data from each community, is 23,059 inhabitants: 16,571 living in Argentina, 4,038 in Bolivia, and 2,450 in Paraguay. These communities are characterized by an important rural and disperse population with a strong presence of aboriginal ethnicities, predominately Wichi, Chorote, Chulupi, and Qom, [4] and multiple healthcare barriers to face in order to obtain high quality medical practices. This includes different cultural patterns [5] and geographical isolation, which tend to be the most relevant barriers. Moreover, centuries of isolation could explain the low demand of healthcare for this population and the consequent lack of basic health infrastructure.

### Characteristics of the local healthcare service

Both Crevaux and D’Orbigny in Bolivia have their own primary healthcare centers, as do the localities of Pozo Hondo and San Agustín/Doctor Pedro P. Peña in Paraguay. In Argentina, there are numerous primary healthcare centers in different rural communities and localities as well as two hospitals located in Alto la Sierra y Santa Victoria Este (Fig 1; Table 1). The referral center for the communities of Bolivia is located in Yacuíba, 150 km away through a dirt road. Pozo Hondo and San Agustín/Pedro P. Peña are dependent on a hospital found in the locality of Mariscal Estigarribia, which is 300 km away on a dirt road. In Argentina, the hospital for the city of Tartagal is the closest to the area, and it is 150 km away from Santa Victoria Este on the only asphalt road in the entire area. Therefore, many of the inhabitants from these communities are rarely able to get to their referral centers on their own, and, in practice, many people from Bolivia and Paraguay cross the border to receive healthcare in Argentina due to proximity of hospitals with greater capacity including hospitalization. Additionally, the public health system in Argentina is universal and is not limited only to nationals or residents. Moreover, due to the different reasons mentioned above, the possibility of having specialized health personnel and access to an ultrasound and laboratory analysis under good laboratory practices are limited and has important variations between the three countries that comprise the area of intervention (Table 1). Another issue to consider is gratuity of the health service, which usually varies between countries. In other words, although the public health systems in Argentina, Bolivia, and Paraguay are completely free of charge for pregnant women and their children, the composition of the services provided are different for each country, and, in Bolivia, it requires certain out-of-pocket payments in order to have access to an ultrasound, for example. Moreover, in some of the localities, ultrasound or laboratory services are not available, and it is up to the patient to cover the costs to travel to an area where this service is provided.

**Table 1:** Characteristics of the different health centers in the different localities within each country with respect to their medical staff, laboratory, and presence of an ultrasound machine.

Country	Locality	Technical Staff (number)	Medical Staff (number)	Laboratory	Ultrasound
Argentina	Santa Victoria Este	Nurses (10)	General practitioner (5)	Yes	Yes
	Alto la Sierra	Nurses (4)	General practitioner (2)	Yes	No
	El Mulato	Nurses (1)	None	No	No
	Bajo Grande	None	None	No	No
	San Miguel	None	None	No	No
	El Bravo	Nurses (1)	None	No	No
	La Esperanza	None	None	No	No
Bolivia	Creveaux	Nurses (4)	General practitioner (3)	No	No
	D´Orbigny	Nurses (2)	General Practitioner (1)	No	No
Paraguay	San Agustín/ Dr. Pedro P. Peña	Nurses (2); Obstetric Technician (1)	General Practitioner (1)	No	No
	Pozo Hondo	Nurses (1)	None	No	No

## Identification of the population to be included and of the professionals required for implementation

Since the framework is centered on the MTCT of HIV, syphilis, HBV, and ChD, the first step for prevention included in the framework is sexual and reproductive health education. After that, implementation needs to be framed within general obstetric care, including the newborn and the postpartum phase. For this, a specialized team made up of a biochemist, an obstetrician and ultrasound technician, a general doctor, and a pediatrician is required.

## Logistical considerations that need to be taken into account for implementation

Due to the geographic and healthcare service characteristics of the area, all actions need to be conducted in the community where the patients live. Therefore, the specialized health team is the one that moves, not the patients. This is so that all the clinical obstetric, pediatric and neonatology controls are performed in the field. Therefore, all the laboratory tests are based on rapid tests for measurement of glucose in blood, hemoglobin, blood group, and factor; serology for HIV, syphilis, HBV, and ChD; rapid detection of *Streptococcus agalactiae* after the 35th week of pregnancy; and ultrasound monitoring with a portable machine.

## Considerations to ensure patient follow-up during implementation

In order to ensure patient follow-up, all of the actions are coordinated by a general coordination team, technical coordinators (biochemist, obstetrician, pediatrician, and data management coordinator), and co-coordinators of the health system according to the jurisdiction (Argentina, Bolivia, and Paraguay). Moreover, two types of actions need to be considered: periodic and continuous actions. Depending on the type of action, different teams of professionals are needed.

### Periodic actions

Periodic actions are performed by the specialized team who works in an intensive manner for a short period of days (i.e., 5 straight days) with the support of other local professionals and health actors in a periodic manner (i.e., every 60 days). In the periodic visits, all the villages with higher populations and more rural populations should be visited in order to perform the following: clinical control of all pregnant women; ultrasound evaluation; serological testing for HIV, syphilis, HBV, and ChD; dosage of hemoglobin, glucose, blood type, and factor; return of results; collection of samples for confirmatory diagnosis in reference centers; treatment and/or pertinent clinical management of the diverse pathologies detected; coordination of referrals to specialized centers as needed; treatment and monitoring of puerperal women and newborns with diagnosis of an infection of vertical transmission; testing and treatment (as per guidelines and norms) of other children, siblings, and/or partners of pregnant women with a diagnosed infection of vertical transmission; training of local personnel; georeference of the households of all pregnant women, puerperal women, and nursing infants in the program; record of a local clinical history; electronic record with online management; and, finally, planning of actions for the following intervention.

### Continuous actions

Continuous actions should be performed in a permanent manner on behalf of the local sanitary agents and should include registration of new pregnant women, follow-up of conducts and treatments put in place during the periodic actions, and online update of clinical records and data. Specific actions related to the different stages of a woman´s reproductive life need to be taken into account and are detailed in Box 2.

**Box 2. Specific actions related to the different stages of a women´s reproductive life that need to be performed in a continuous and permanent manner by the local sanitary agents and/or healthcare providers**

During pregnancy

Pregnant women need to be actively sought out through the local sanitary or community agents in order to promote early and universal access to prenatal healthcare. The objective is for local professionals to start organizing appointments so that the women are seen by the specialized team during the periodic actions. All pregnant women need to have prenatal controls based on national guidelines.

- Serological screening for the detection of infection by HIV, syphilis, HBV through the HBsAg, and ChD, according to the trimester of pregnancy they are in and following national algorithms. Initially, rapid tests are performed, and those with positive results are confirmed by quantitative methods.
- Depending on the previous vaccination records of the pregnant woman, the vaccination calendar is updated. In Argentina, Bolivia, and Paraguay, a complete vaccination schedule includes a dose at birth within the first 12 hours of life and a booster dose at 2, 4, and 6 months of life.
- Treatment and follow-up of those pregnant women with positive serology for the EMTCT Plus infections
  - Women with HIV infection receive standard antiretroviral treatment.
  - Women with syphilis receive treatment with benzathine penicillin.
  - Women with HBV infection are derived to a regional infectious disease service for evaluation and treatment group.
  - Women with simultaneous infection with HIV and HBV receive antiretroviral treatment.
  - Women with *Trypanosoma cruzi* infection are monitored until finalization of the pregnancy in order to organize their treatment following delivery as well as diagnosis and treatment of the newborn.

Prenatal and postnatal period

Newborns from HIV infected mothers receive prophylaxis according to current national norms during the first six weeks of birth. Nursing infants exposed to HIV will be tested for infection through the use of a blood PCR between the fourth and sixth week of birth, and a second sample will be taken and analyzed if the first sample was positive in order to confirm the diagnosis. Additionally, a rapid serological diagnosis test will be used for the detection of HIV antibodies at 18 months of life. All children infected with HIV will receive integral medical attention through referral to their reference center.

Nursing infants with symptoms compatible with MTCT of syphilis or exposed to the risk of infection will be treated and monitored clinically and serologically in order to confirm seroconversion. Nursing infants of HBsAg-positive mothers will be evaluated for the presence of specific antigens for one to three months after completing the vaccination scheme. Additionally, the vaccination scheme against hepatitis B will be completed using the pentavalent vaccine following the national vaccination calendars: at 2, 4, and 6 months of age with a minimum interval of 4 weeks in between doses. Newborns of mothers’ positive for HBsAg will receive immunoglobulin specifically against hepatitis B (100 IU) in the first 12 hours after birth. All newborns will also receive the HBV vaccine in the first 12 to 24 hours after birth using the monovalent vaccine as per the national vaccination calendars.

Newborns of *T*. *cruzi* infected mothers receive parasitological screening for the presence of the parasites through the use of a PCR in the postnatal period [7–11]. Newborns with positive PCR results start treatment. After birth, mothers receive counseling on family planning and contraception, and treatment with benznidazole is provided to those with positive serology for *T*. *cruzi* [12]. Those newborns of infected mothers with negative parasitology for *T*. *cruzi* in the postnatal period will be evaluated serologically for the presence of specific antibodies against the parasite at 10 months of age, when the maternal antibodies have waned. Children with positive serology for *T*. *cruzi*, will be treated with benznidazole before the first year of age, and their clinical management with serological monitoring will be performed as per national guidelines [6].

## Management of patient data for implementation

For the compilation of data and for them to be able to be used in an agile manner in order to be able to make decisions when seeing a patient, software that allows working in an offline manner was developed. This software is an open-source software called MySQL (Oracle Corporation), which is a type of relational database management system (RDBMS) that may be adapted to other contexts. Tablets are also used to allow for mobility and working in remote areas without connectivity. Recorded data are geolocalized in each tablet and then synchronized to a server that centralizes the information and allows online access from any device. Each operator has a username and password to access the system with different authorization levels, allowing either the viewing of clinical histories with follow-up visits and test results, the viewing and recording of data, or the viewing and recording of laboratory results. Moreover, the software allows the use of filters in order to be able to search for specific patients grouped by community, geographical area, age, pathology, and others and to be able to perform basic statistics. All actions are recorded in real time through tablets that are available at the sites where medical assistance is provided. In the same manner, all the data from complementary evaluations are entered either at the site or from the laboratories that perform the confirmatory tests. Data are accessible for project coordinators depending on the levels of assistance, and they are confidential. Moreover, different profiles of access are programmed so that depending on the role in the project, only certain parts of the data are available. For example, the authorities from the different countries only have access to the data from their own country.

## Other actions which need to be considered for implementation within the framework

Due to the characteristics of the infections included in the framework, certain transversal actions need to be implemented. For infection by HIV, tests are offered for sexual partners and previous children of a seropositive woman. Positive individuals are referred to the closest jurisdictional greater complexity hospital for treatment and follow-up. For infection by HBV, a test for the detection of HBV (HBsAg) for sexual partners, children, siblings, and/or other family members and direct household contacts of people with a positive result should be conducted. Individuals with negative serology for HBAgs should be vaccinated as well as those that have never been vaccinated. For syphilis infection, treatment with benzathine penicillin (2.4 million units in a single dose, intramuscularly) for early syphilis or benzathine benzylpenicillin (2.4 million units weekly, intramuscularly) for three consecutive weeks for late syphilis or unknown stage should be given to all the partners of women positive for the infection. Transversal actions for ChD should include promotion measures to achieve the interruption of vector transmission in households and serological diagnosis of all previous children of pregnant women detected positive for *T*. *cruzi* infection. Any complex case that cannot be resolved in each locality is referred to the jurisdictional hospital with greatest complexity. This is the hospital of the city of Tartagal (160 km) for Argentina, the hospital of Mariscal Estigarribia (300 km) for Paraguay, and the hospital of Yacuíba (150 km) for Bolivia.

## Conclusion

The protocol of a program centered on pregnant women and newborns is presented for the Tri-Border Area of Argentina, Bolivia, and Paraguay in the Gran Chaco region. This protocol has been implemented since June 2018 and has shown that it is feasible to undertake a public–private initiative in areas of high social and geographical vulnerability in three different countries with varying healthcare systems (Table 2 shows preliminary coverage data from the first year of implementation). This first implementation phase has also shown that it is possible to offer quality healthcare practices when there are tailor-made programs that specifically evaluate and address the needs of the target population. Finally, this program provides evidence that in order to be able to reach the goals of the EMTCT Plus framework, national strategies need to contemplate and adapt to local realities. The idea is for this project to serve as a model that may be implemented in other areas; although in order for the current project to be sustainable, one strategy that will be used in the second phase of the project is to incorporate and train regional medical doctors that can eventually take over the activities of the professional staff of the project. These would be professionals from Salta (Argentina) as well as from Bolivia and Paraguay. Furthermore, the local staff that is currently implementing the continuous activities is already trained and capable of continuing with these actions once the project is finalized. Ultimately, we hope that the information provided in this symposium piece is useful for implementation of the framework in similar areas.

**Table 2:** Preliminary data from the first year of implementation.

Country	Census population	Estimated number of pregnancies	Number of controlled pregnancies^1^ through implementation	Coverage of pregnancies
**Argentina**	16,571	392^2^	485	124%
**Bolivia**	4,038	89^3^	79^4^	88.8%
**Paraguay**	2,450	41	47	114%
**Total**	23,059	522	611	117%

^1^At least one complete evaluation was performed (including laboratory tests and ultrasound), since at the start of the project there were women in different trimesters of pregnancy.

^2^Data obtained from notified pregnancies for the specific area in 2017, prior to the start of the project.

^3^Number of pregnancies was estimated by using the birth rate for Bolivia and Paraguay (average annual number of births during a year per 1,000 persons in the population at midyear) from the Central Intelligence Agency factbook since official data from the specific areas is not available: https://www.cia.gov/library/publications/the-world-factbook/rankorder/2054rank.html.

^4^Two of the planned periodic visits could not be performed due to inclement weather.

**Key Learning Points**

- Adding quality actions for prenatal control in the context of the EMTCT Plus framework made its implementation more effective and efficient.
- In order to implement the EMTCT Plus framework in dispersed populations that do not have the means to get to a referral center on their own or do not live in areas with local specialized health personnel, it is fundamental to rely on a local team (not necessarily highly trained) for patient uptake and follow-up.
- Counting with point-of-care diagnostics is imperative in order to be able to take immediate actions related not only to the patient but also to the family depending on the epidemiological situation (i.e., study of partners for sexually transmitted diseases or study of other children in the case of ChD).
- In remote areas, the adaptation of certain diagnostic and treatment norms is sometimes required in order to be able to adapt them to the local situation in which the distance to a referral center is not contemplated.
